# Emerging Approaches for Fluorescence-Based Newborn Screening of Mucopolysaccharidoses

**DOI:** 10.3390/diagnostics10050294

**Published:** 2020-05-11

**Authors:** Rajendra Singh, Shaileja Chopra, Carrie Graham, Melissa Langer, Rainer Ng, Anirudh J. Ullal, Vamsee K. Pamula

**Affiliations:** Baebies, Inc., P.O. Box 14403, Durham, NC 27709, USA; samachargaya@gmail.com (R.S.); shaileja.chopra@gmail.com (S.C.); cgraham@baebies.com (C.G.); mlanger@baebies.com (M.L.); rng@baebies.com (R.N.); aullal@baebies.com (A.J.U.)

**Keywords:** fluorescence, newborn screening, lysosomal storage disorders, mucopolysaccharidoses

## Abstract

Interest in newborn screening for mucopolysaccharidoses (MPS) is growing, due in part to ongoing efforts to develop new therapies for these disorders and new screening assays to identify increased risk for the individual MPSs on the basis of deficiency in the cognate enzyme. Existing tests for MPSs utilize either fluorescence or mass spectrometry detection methods to measure biomarkers of disease (e.g., enzyme function or glycosaminoglycans) using either urine or dried blood spot (DBS) samples. There are currently two approaches to fluorescence-based enzyme function assays from DBS: (1) manual reaction mixing, incubation, and termination followed by detection on a microtiter plate reader; and (2) miniaturized automation of these same assay steps using digital microfluidics technology. This article describes the origins of laboratory assays for enzyme activity measurement, the maturation and clinical application of fluorescent enzyme assays for MPS newborn screening, and considerations for future expansion of the technology.

## 1. Introduction

Mucopolysaccharidoses (MPS) are a collection of at least 11 rare genetic disorders that are progressive and vary widely in severity. Most affected children appear normal at birth, with symptoms (including coarse facial features; skeletal and joint changes with limitation in movement; and abnormalities in the liver, spleen, heart, and blood vessels) first developing around or after their first birthday. Patients with MPS require timely diagnosis and treatment for better outcomes. Although individually rare (1:40,000 to 1:200,000), the combined incidence of all MPS disorders is around 1 in 25,000 [1]. Tomatsu et al. estimate that as many as 200 babies are born each year in the United States that are affected with one type of MPS [2]. Although rare, these disorders are devastating to children and their families [3].

**Figure 1:**
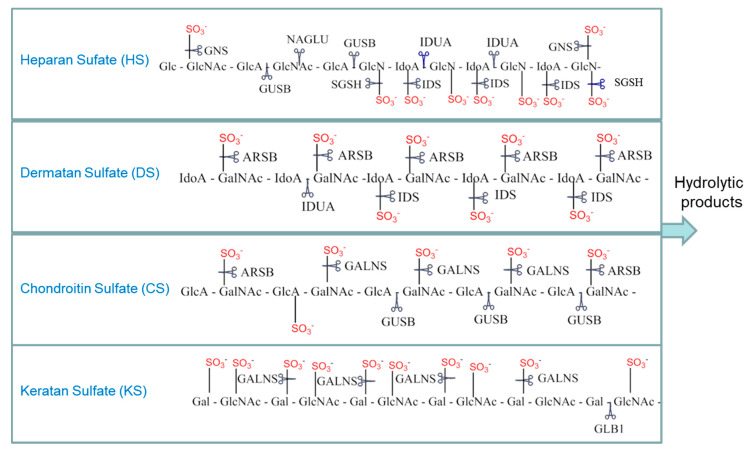
Select examples of glycosaminoglycan breakdown by the mucopolysaccharidoses (MPS) enzymes. Scissors indicate the sites cleaved in each of the glycosaminoglycans (GAGs) by the various MPS enzymes. HGSNAT (not shown) is a transferase that adds an acetyl group to glucosamine residues. GUSB also cleaves dermatan sulfate after GlcA residues are exposed by other enzymes. Gal: galactose; GlcA: glucuronate; IdoA: iduronate; GlcNAc: *N*-acetylglucosamine; GalNAc: *N*-acetylgalactosamine; IDUA: α-iduronidase; IDS: α-iduronide sulfatase; SGSH: *N*-sulfoglucosamine sulfohydrolase; NAGLU: acetyl α-glucosaminidase; GALNS: *N*-acetyl galactosamine-6-sulfatase; GLB1: β-galactosidase; ARSB: *N*-acetyl galactosamine 4-sulfatase; GUSB: β-glucuronidase; GNS: *N*-acetyl glucosamine-6-sulfatase; HGSNAT: β-glucosaminide *N*-acetyl transferase.

The 11 recognized types of MPS are each caused by the deficiency of a specific lysosomal enzyme, which results in the progressive accumulation of glycosaminoglycans (GAGs) in tissues throughout the body and produces the clinical phenotype associated with MPS [3,4,5]. GAGs are heteropolysaccharides composed of repeating disaccharide units with a high negative charge due to the presence of multiple carboxyl groups and sulfate substitutions. The GAGs elevated in MPSs include dermatan sulfate (DS), heparan sulfate (HS), keratan sulfate (KS), and chondroitin 6-sulfate (CS) (Figure 1). Hyaluronic acid is a fifth GAG species that is elevated only in mucopolysaccharidosis type IX (also known as Natowicz syndrome and hyaluronidase deficiency).

Clinical phenotypes of the various MPSs (summarized in Table 1) differ on the basis of the specific enzyme deficiency, but usually include facial dysmorphism, hepatosplenomegaly, cardiac, respiratory, and skeletal involvement, and neurological, hematological, and ocular symptoms. Not included in Table 1 is MPS IX, an extremely rare form of the disease that was first described in 1996 [6] and is not yet well characterized. 

## 2. Overview of Newborn Screening for MPS Disorders

Newborn screening (NBS) was first introduced in the United States in the 1960s with a single test for phenylketonuria (PKU), an inherited metabolic disorder that causes elevated levels of phenylalanine in the blood and severe intellectual impairment but that can be effectively treated if identified soon after birth. NBS has subsequently expanded across the United States to include dozens of tests and approximately 45 distinct conditions [7]. All but two of these conditions (congenital hearing loss and critical congenital heart disease) are tested in the public health laboratory setting using a single, small volume blood sample—a dried blood spot (DBS), which is collected by capillary draw from heel-stick of the newborn and dried onto filter paper. This filter paper is also commonly referred to as a “Guthrie card” after Dr. Robert Guthrie who developed the newborn DBS collection technique for PKU screening [8]. DBS offers many advantages over whole blood or isolated leukocyte samples, including simple collection methodologies, ease of transport, and excellent stability for long-term storage [9]. The expansion of NBS has been fueled by the introduction of new therapies, new testing methods, and new technologies [10]. Most notably, the implementation of tandem mass spectrometry (MS/MS) by Millington and colleagues in the early 2000s enabled dozens of small molecule metabolites (<1000 Da in molecular weight, e.g., amino acids and acylcarnitines), indicative of increased risk of over 30 different conditions, to be analyzed using a single punch of the DBS sample (approximately 3.2 mm) [11]. In contrast to this metabolite-based NBS approach, NBS assays for MPSs and other lysosomal storage disorders rely on direct measurement of enzyme activity to detect enzyme deficiency. 

NBS for MPS I (Hurler/Scheie) was recommended for inclusion on the U.S. Recommended Uniform Screening Panel (RUSP) in 2016 [12], and at the time of writing, 21 states (California, Delaware, Illinois, Kentucky, Maryland, Massachusetts, Michigan, Minnesota, Missouri, Nebraska, New Jersey, New York, Ohio, Oregon, Pennsylvania, Rhode Island, Tennessee, Vermont, Virginia, Washington, and Wisconsin), the District of Columbia, and several programs outside of the United States (Taiwan, Italy, and others) screen for this condition [13]. On the basis of the rate of implementation of other conditions recently added to the RUSP, it is anticipated that screening for MPS I will be conducted in all state programs within the next 10 years. 

Additional MPSs are expected to be added to the RUSP when reliable screening tests and treatments become available. MPS II (Hunter) is another candidate for widespread NBS and is expected to be nominated for inclusion on the RUSP soon. Two states (Illinois and Missouri) and some programs outside of the United States (Taiwan) have already begun screening for MPS II [13,14]. Effective enzyme replacement therapies are currently available for many of the MPSs, including MPS I, MPS II, MPS IVA (Morquio A), MPS VI (Maroteaux–Lamy disease), and MPS VII (Sly) [15,16]. As more therapies are developed for the other MPSs, demand for complementary screening assays to identify affected newborns is also anticipated to grow. 

## 3. Historical Overview of Laboratory Methods for Enzyme Activity Measurement

The analysis and reporting of enzyme activity is highly dependent on the type of sample used, such as fibroblasts, leukocytes, plasma, or DBS. DBS specimens are the preferred samples in NBS public health laboratories, and consequently, this type of sample is utilized in all current MPS newborn screening assays. 

**Figure 2:**
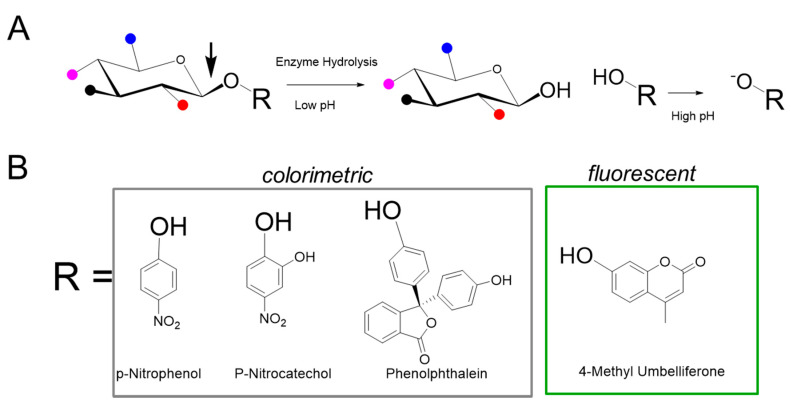
Synthetic substrates for MPS enzyme measurement. (**A**) The generic structure of the substrate is composed of a specific saccharide, conjugated to an aglycone (R group). Various functional groups are indicated by colored balls, for example red for sulfate, and are attached to the sugar. The enzyme of interest cleaves the glycosidic bond denoted by the arrow to release R, which is then exposed to high pH conditions to generate signal. (**B**) Examples of aglycone moieties typically used in colorimetric and fluorescence based assays.

Historically, in vitro measurement of enzyme activity was performed using natural substrates with radioactive labels, which were quantified on the basis of the amount of product generated by enzymatic cleavage of the substrate. These methods had multiple steps with hazardous radioactive substrates and required the separation of radiolabeled product from substrates; nevertheless, they served as the foundation for high throughput nonradioactive analytical methods for enzyme measurement. The synthetic substrates used in nonradioactive glycosidic assays are conjugates of a sugar appended to an aglycone structure (Figure 2) and are sometimes referred to as artificial because the aglycone moiety is not present in the natural substrate. The sugar component of the substrate is either the natural sugar that is a substrate for the particular enzyme or a modified sugar that maintains function sufficient to be a substrate for the particular enzyme to be assayed. The aglycone component of the substrate, when cleaved from the substrate, allows for analysis of the enzymatic activity by optical and mass spectrometric methods [17,18]. The fluorimetric substrates used to interrogate sulfatases, 4-methylumbelliferyl sulfate (4-MUS) for example, typically do not include a sugar and instead attach the fluorimetric leaving group directly to the sulfate. Although various sulfate cleaving enzymes have traditionally been classified as arylsulfatases, the actual sulfate in the natural substrate is not on an aryl group (e.g., 4-MUS).

Van Hoof and Hers first described a glycosidase activity measurement method to detect abnormalities of lysosomal enzymes in MPS using glycoconjugate substrates composed of chromophores such as phenolphthalein, p-nitrophenol (p-NP) [19], and paranitrocatechol (pNC) [20]. Cleavage of the substrate liberates phenolphthalein to produce a purple color or nitrophenolate to produce an intense yellow color (absorbance 405 nm). Such colorimetric labels derived from acid base indicators either require alkaline conditions or oxidation to exhibit the chromophoric color change. Importantly, the aglycone product of the enzymatic reaction can be detected in the presence of the conjugated glycoside substrate, thereby obviating the need for specialized equipment to separate the product from the substrate. Aglycone labels such as p-NP, pNC, phenolphthalein, and indoxyl derivatives have been used for interrogation of multiple hydrolytic enzymes including phosphatases, sulfatases, and glycosidases. The use of chromophores derived from p-NP and pNC is somewhat limited by the sensitivity of absorbance, particularly in miniaturized systems with shorter path lengths. Colorimetric substrates absorbing approximately 400–550 nm are also prone to spectral interference with blood samples, which renders the method incompatible with DBS samples.

## 4. Fluorescent Enzyme Activity Measurement

Fluorescent assays for enzyme activity measurement were first described in the 1950s and have superior sensitivity compared to colorimetric enzyme reactions. The improved sensitivity achieves lower limits of detection—a critically important factor in determining low concentrations of enzymes in complex samples such as DBS. For example, coumarins—a class of lactones comprising fused benzene and α-pyrone rings (benzopyrones)—have been easily adapted as enzyme substrates [21,22,23]. They are widely used as small-molecule fluorophores due to their high quantum yield, large Stokes shift, cell permeation, ease of preparation, and excellent solubility. Coumarin-based fluorescent probes have been used in several applications, including interrogation of enzymatic activities and metal sensing [24,25].

The most widely used coumarins are the 4-methylumbelliferyl derivatives (4-MU). Mead and co-workers pioneered the use of 4-MU substrates in enzymatic assays [26], and made the important discovery that the hydrolytic products of umbelliferone glucuronides (hydroxycoumarins) require high pH conditions (pH 10–11) for greatest fluorescence. Shortly thereafter, various derivatives of 4-MU were investigated as fluorogenic substrates for a range of glycosidase enzymes in bacterial samples [27,28]. Methyl umbelliferyl-based substrates have been developed for nearly all the MPS disorders and tested against various samples under myriad conditions (see Table 2). Some of the MPSs require two-step reactions where the MPS enzyme cleaves a functional group in the first step and another enzyme, then liberates the fluorescent 4-MU product. In some instances, both these steps are combined into a single reaction [29].

Stirling et al. (1978) and Hopwood et al. (1979) described the first assay of α-l-iduronidase (IDUA) in leukocytes and cultured fibroblasts using 4-MU-α-l-iduronide as the substrate [30,31]. These fluorescent assays were able to discriminate deficient and normal enzyme activities in samples obtained from MPS I patients and controls, respectively. Minami et al. compared the activities of two different substrates—a colorimetric phenyl-α-l-iduronide to a fluorescent 4-MU-α-l-iduronide using leukocytes and lymphoblastoid cells obtained from patients with MPS I Hurler and Scheie syndromes [32]. They found increased sensitivity in the 4-MU substrate, which translated to shorter incubation times.

More recently, the aglycone moieties in fluorescent substrates have been utilized as mass tag labels for mass spectrometry analysis [33]. The MS/MS approach uses an internal standard that is typically the heavier deuterated analog of the cleaved aglycone and is used along with the substrate (internally) to correct for any loss of signal. The deuterated internal standard is needed to quantify the enzyme activity from the parent mass-to-charge ratio (*m*/*z*) to daughter fragmentation. Lee et al. demonstrated a one-step direct assay for α-iduronide sulfatase (IDS) using ultra performance liquid chromatography (UPLC)–MS/MS and commercially available fluorescence reagents, that is, the 4-MU-based IDS substrate and a 4-MU-based glucose internal standard [34].

## 5. Fluorescent Assays for MPSs

Seminal work from Chamoles and coworkers [35,36,37] demonstrated that activities of hydrolytic enzymes can be measured from DBS samples using 4-MU-linked fluorescent synthetic (“artificial”) substrates. This work paved the way for the first use of fluorescence-based enzyme activity assays for MPS I NBS in Taiwan beginning in 2008 [38]. Fluorescent enzyme assays for IDUA and α-iduronide sulfatase (IDS), the enzymes deficient in MPS I and MPS II, respectively, have also been miniaturized and automated by Sista et al. [39,40] using digital microfluidics (DMF) technology. The IDUA DMF fluorescent (DMF-F) assay was cleared by the FDA in 2017 for newborn screening using DBS samples [41], and DMF-F assays for several other MPS enzymes are in development.

**Figure 3:**
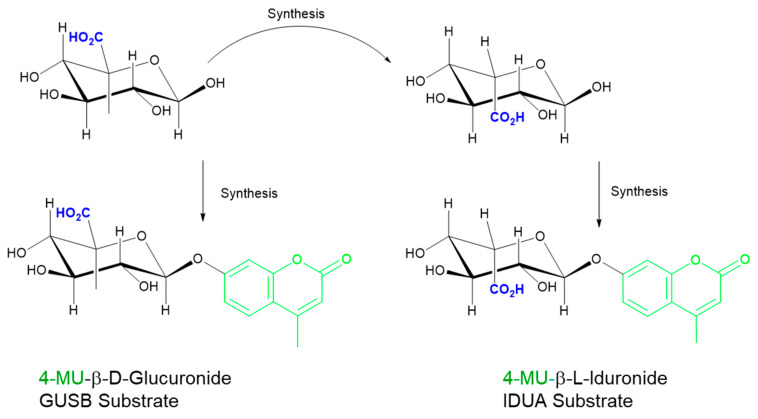
Importance of stereochemistry for specificity. Iduronide (top right) is derived from the stereochemical inversion of the CO_2_H group at C5 in glucuronide (top left). Incomplete inversion leads to glucuronide (GlcA) presence in the iduronide (IdoA) substrates and necessitates the use of inhibitors to suppress glucuronidase activity. GUSB: β-glucuronidase; IDUA: α-iduronidase.

Two general approaches are currently utilized in fluorescent assays to interrogate MPS enzyme deficiencies: one-step or multi-step (sequential) reactions. Both use synthetic substrates that must be of the highest structural specificity through stereochemistry (e.g., iduronide for MPS I and glucuronide for MPS VII; Figure 3) and require specialized assay conditions (*k_cat_*/*K*_M_, pH, inhibitors, buffer system, and other reaction components) for optimal assay performance. With these existing assay formats, each enzyme must be measured individually using physiologically specific conditions. Additionally, a novel approach for pan-MPS enzyme measurement that simultaneously quantifies five of the MPS enzymes using fluorescent analysis of glycosaminoglycans (GAGs) has been developed and will be discussed.

### 5.1. One-Step Hydrolysis Assays

The enzymes deficient in MPS I, MPS IIIB, MPS IVB, and MPS VII are assayed in single-step hydrolytic reactions incorporating either a kinetic or endpoint detection format. When using the non-specific 4-MUS substrate, MPS VI can be interrogated using a single-step reaction; however, this method is not optimal, as described in Section 5.2. The fluorescent substrate used in each of these assays is composed of an aglycone dye (4-MU) bound to the reaction-specific sugar by a glycoside bond (Figure 2). Under acidic conditions (pH 3–5.5), the endogenous enzyme in the DBS sample hydrolyzes the glycosidic bond of the substrate to produce umbelliferone proportional to the level of enzyme activity in the sample. In a kinetic assay, the reaction is read at various time points after termination of the enzymatic reaction. Termination is usually performed at a high pH (pH 10–11) and serves two purposes—to stop the enzymatic reaction and simultaneously to deprotonate the phenolic group on the umbelliferone to enhance its fluorescence. The pKa of the phenolic group in umbelliferone is 7.8; under the acidic conditions used in the assay incubation, the liberated, protonated umbelliferone is relatively non-fluorescent [42,43].

Substrate purity has been a challenging factor for MPS assay optimization and standardization. In the case of IDUA, because it is difficult to detect trace amounts of the non-desired anomer in the synthetic substrate (<1%), d-saccharic acid-1,4-lactone should be added to block the action of beta-glucuronidase in the assay. The substrates of MPS I and MPS VII differ in the stereochemistry at the C5, as well as the anomeric carbon where the aglycone is attached (Figure 3). The inhibitor 1-4 saccharolactone was initially used at high concentrations to suppress the glucuronidase activity [31,32], however, recent commercial sources of iduronide have significantly improved purity [32] and therefore achieve specificity at lower saccharolactone concentrations (unpublished results).

The fluorimetric newborn screening assays for MPS IIIB, MPS IVB, MPS VI, and MPS VII (illustrated in Figure 2) are also based on single step reactions [44,45]. MPS VII (β-glucuronidase (GUSB) deficiency) can be identified using a single step fluorescent reaction with the 4-MU glucuronide substrate [46]. For MPS IIIB, the substrate, 4-methylumbelliferyl-2-acetamido-2-deoxy-α-d-glucopyranoside, is utilized, and enzymatic cleavage liberates fluorescent 4-MU upon N-acetylglucosaminidase (NAG)-mediated cleavage of glycoside. A fluorescent assay suitable for MPS IVB newborn screening uses the substrate 4-methylumbelliferyl-β-d-galactoside (4-MUG) in a single step reaction to measure galactosidase-β-1 (GLB1) activity [47,48]. However, deficient GLB1 activity is also found in another lysosomal storage disease (GM1 gangliosidosis), which is not classified as a MPS and, therefore, an identification of GLB1 deficiency alone cannot discriminate between these two conditions. An orthogonal measurement of GAGs is recommended to delineate between MPS IVB and GM1 gangliosidosis [47]. All of these single-step enzyme assays have been published using standard multiwell plate fluorimetry [44,45,46,47] and have recently been automated on the DMF-F platform [49].

### 5.2. Two-Step/Sequential Reactions

Several of the MPSs are caused by deficiencies in enzymes that must be assayed using more complex methods in which a second step or a coupled enzyme reaction generates fluorescence. MPS II, MPS IIID, MPS IVA, and MPS VI are caused by deficiencies in sulfatases (IDS, *N*-acetyl glucosamine-6-sulfatase, *N*-acetyl galactosamine-6-sulfatase, and *N*-acetyl galactosamine 4-sulfatase, respectively); MPS IIIA is caused by deficiency in a sulfamidase (*N*-sulfoglucosamine sulfohydrolase; SGSH); and MPS IIIC is caused by deficiency in an acetyl transferase (α-glucosaminide *N*-acetyl transferase; HGSNAT). Each of these reactions is described in further detail in this section.

**Figure 4:**
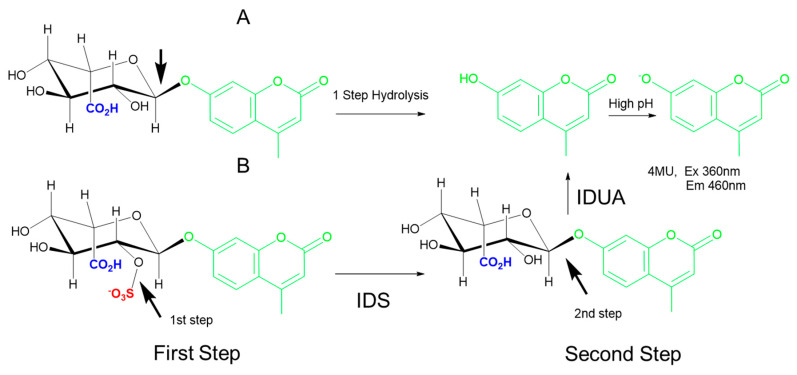
Examples of one-step and two-step hydrolysis reactions. (**A**) The one-step hydrolysis of the 4-methylumbelliferyl derivative (4-MU)-IdoA substrate cleaves the glycosidic bond indicated by the arrow to liberate 4-MU, which is measured under high pH conditions. (**B**) The two-step hydrolysis of 4-MU-IdoAS requires cleavage of the red sulfate first (arrow in left bottom) to yield 4-MU-IdoA. In the second step of the reaction (bottom right), IDUA cleaves the glycosidic bond indicated by the arrow to liberate 4-MU, which is measured under high pH conditions. IDS: α-iduronide sulfatase; IDUA: α-iduronidase.

Interrogation of IDS (a sulfatase, not a glycosidase) requires two steps: a first step to cleave the sulfate group attached at the C2 of the iduronic acid, followed by a sequential hydrolysis step to cleave the glycosidic bond and liberate the 4-MU aglycone (Figure 4) [50,51,52]. Early fluorimetric protocols for IDS measurement were relatively slow, labor intensive, and expensive due to the requirement for a costly substrate and the ancillary IDUA enzyme; improvements in substrate manufacturing and miniaturization through DMF have significantly reduced materials costs. Recent studies have also shown that it is possible to combine the IDUA and IDS substrates in a single reagent such that the sequential reactions take place concurrently [29]. A modified version of this fluorimetric enzyme measurement assay [51] with a novel stop buffer is currently in use for MPS II newborn screening in the state of Missouri and provides results from DBS in around 2 hours [14].

*N*-acetylglucosamine-6-sulfatase (GNS), the deficient enzyme in MPS IIID, is another sulfatase that cleaves the sulfate from *N*-acetylglucosamine-6-sulfate residues in heparan. The earliest assays for GNS activity utilized heparan-derived radiolabeled oligosaccharides [53,54]. He et al. first described the use of a fluorimetric substrate (4-MU-α-*N*-acetyl glucosamine-6-sulfate) for GNS measurement using fibroblast and leukocyte samples [55]. Liberation of the 4-MU from this substrate requires two enzymes in a sequential fashion: GNS, followed by *N*-acetyl glucosaminidase. The latter is added exogenously in a second incubation, as reliance on endogenous levels is insufficient to develop a robust assay with short incubation times. To our knowledge, there are no published methods for GNS measurement that are compatible with DBS samples.

**Figure 5:**
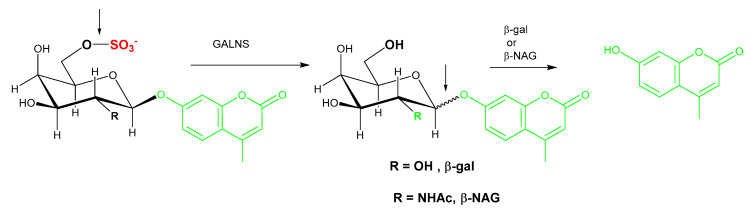
Illustration of the two-step GALNS reaction. In the first step of the reaction, the 4-methylumbelliferyl-β-d-galactopyranoside-6-sulfate substrate is cleaved by GALNS to remove the sulfate group and produce 4-methylumbelliferyl-galactoside. Exogenous β-gal or β-NAG is required to liberate the fluorochrome from 4-MU. GALNS: *N*-acetyl galactosamine-6-sulfatase; β-gal: β-galactosidase; β-NAG: β-*N*-acetyl-glucosaminidase.

Similarly, the assay for *N*-acetylgalactosamine 6-sulfatase (GALNS), the deficient enzyme in MPS IVA uses 4-methylumbelliferyl-β-d-galactopyranoside-6-sulfate as the substrate for enzymatic cleavage of the C-6 sulfate in the first step, followed by cleavage of the glycosidic linkage in the second step (Figure 5). The galactose-6-sulfate based substrate has been used to assay GALNS activity in leukocytes [56] and DBS [57]. The enzymatic liberation of the fluorochrome from 4-MU substrate requires the sequential action of galactose-6-sulfate sulfatase and β-galactosidase. Endogenous β-galactosidase activity is able to completely hydrolyze the non-fluorescing 4-methylumbelliferyl-galactoside formed in the first step during incubation. However, a second incubation in the presence of excess β-galactosidase is needed to avoid underestimation of galactose-6-sulfate sulfatase activity as the endogenous β-galactosidase may be low in certain samples. Van Diggelen et al. analyzed fibroblasts and leukocytes from 12 different MPS IVA (Morquio A) patients and found 0.0%–2.7% of mean normal galactose-6-sulfate sulfatase activity, with heterozygotes showing intermediate activity [56].

Substrate purity is a noted issue for two-step enzyme assays as, in addition to the aglycone substrate for the enzyme of interest, the substrate for the second enzyme could be present as a contaminant. For GALNS activity measurement, commercially available sources of 4-MU-galactose-6-sulfate provide a convenient option, however, endogenously present β-galactosidase and hexosaminidase also cause additional non-specific hydrolysis of 4-MU-galacatose (Figure 5, R=OH) and 4-MU-N-acetyl-galactose (Figure 5, R=NHAc), respectively. The *N*-acetylated substrate 4-MU-*N*-acetyl-gal-6-sulfate is a superior alternative substrate as it obviates the background signal, however, an additional inhibitor of hexosaminidase A (Z)-Pugnac [O-(2-Acetamido-2-deoxy-D-glucopyranosylidene) amino-Z-*N*-phenylcarbamate] and a second bacterial enzyme (β-*N*-acetyl-galactosaminidase) are needed [53]. The lack of a commercial source of β-*N*-acetyl-galactosaminidase is a barrier to widespread uptake of this assay.

**Figure 6:**
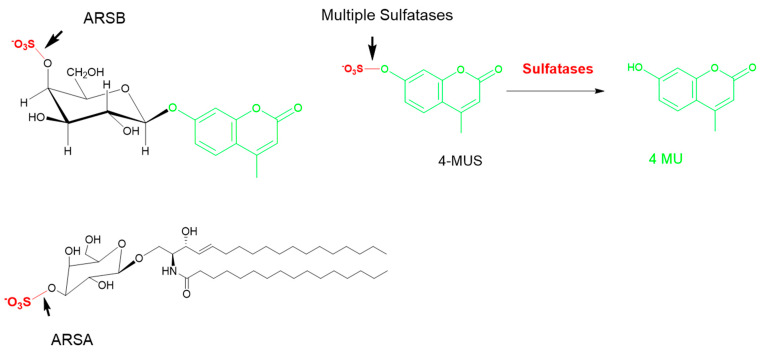
The common methylumbelliferyl sulfate (MUS) substrate. Multiple sulfatases are able to cleave the sulfate group in 4-MUS, as the only commonality is the sulfate group. Examples of specific substrates for ARSA (cerebroside galactosyl sulfate) and ARSB (GalS) are shown on the left. ARSA: arylsulfatase A; ARSB: *N*-acetyl galactosamine 4-sulfatase.

The fourth sulfatase relevant for MPS etiology is *N*-acetyl galactosamine 4-sulfatase (ARSB), which cleaves a sulfate group from the C4 of an *N*-acetyl galactose or galactose moieties in GAGs. The synthetic substrate is either 4-MU–galNAc-4-sulfate or 4-MUS, the latter being a very nonspecific substrate that only has the sulfate group in common and is cleaved by multiple sulfatases (Figure 6). The arylsulfatase nomenclature was derived from the fact that the enzymes cleaved a sulfate group from an aromatic group, although the natural substrate usually is a sulfate on an aliphatic group, usually a sugar. The advantages and drawbacks of the specific and non-specific substrates will be discussed in further detail later in this article. 

**Figure 7:**
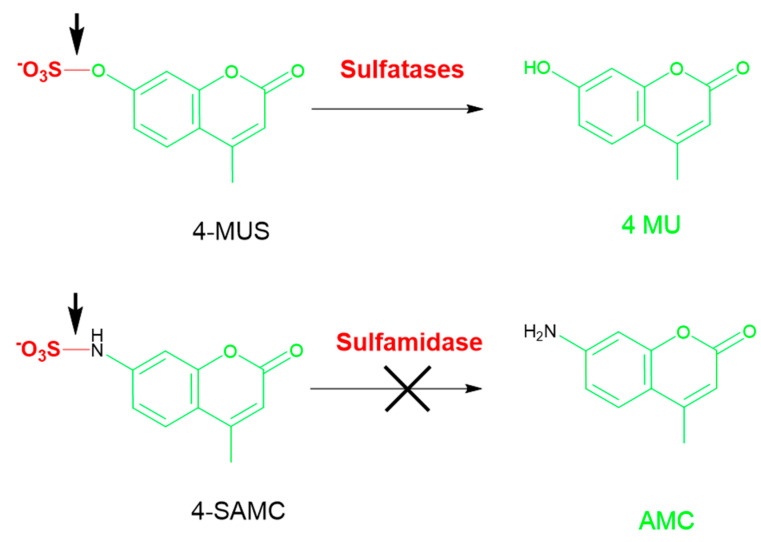
4-MUS with an O–SO_3_ bond was used as a generic substrate for multiple sulfatases (top). SGSH fails to cleave a generic substrate SAMC substrate with a N–SO_3_ bond incorporating the common fluorophore 4-MU (bottom). AMC: amidomethylcoumarin; SAMC: sulfamidomethylcoumarin.

MPS IIIA is caused by a deficiency in sulfamidase (SGSH). The fluorescence-based assay for SGSH, described by Karpova et al. uses a 4-methylumbelliferyl-α-D-*N*-sulfoglucosaminide (4-MU-α-GlcNSO_3_ substrate) [58]. In this two-step reaction, SGSH cleaves the *N*-sulfate of the 2-sulfamino-2-deoxy-d-glucopyranosyl residue to generate 4-MU-αGlcNH_2_. The substrate is commercially available, but requires careful characterization to ensure enantiomeric and chemical purity. Assays measuring SGSH activity have been established using skin fibroblasts and leukocytes [58], cell lysates, and tissue homogenates [59,60,61], with each sample type requiring distinct assay conditions for optimal performance. Several approaches have attempted to develop a fluorescent SGSH assay for DBS, however, none have met the performance requirements necessary to support NBS yet [62]. In particular, the MPS IIIA assay for SGSH using the umbelliferyl α-glucosamine that is *N*-sulfated could not be desulfated under a variety of conditions using DBS. Gelb et al. have explored modification of the key saccharide(s) as well as the aglycone moiety in order to optimize the biochemical parameters *K*_M_ and *V*_max_ for a viable DBS assay. A napthol-based aglycone with the α-glucosamine *N*-sulfamide yielded the best separation with quality control (QC) DBS samples [63]. We also attempted to develop a fluorescent SGSH assay suitable for DBS using a custom 4-MU based sulfamidomethylcoumarin (SAMC) substrate with an N-sulfate that was envisioned to be cleaved by sulfamidases, but with greater specificity than previous 4-MUS substrates that cleave *O*-sulfate (illustrated in Figure 7). The SAMC substrate, however, failed to liberate 4-MU under a myriad set of conditions (unpublished results). 

Thus, both the saccharide as well as the aglycone moiety are needed to accurately quantify activity from sluggish enzymes and in samples such as DBS with a low abundance of enzyme. The failure of SAMC as well as MUS with SGSH highlights that these substrates, although easily available, are not appropriate for the most recalcitrant enzymes.

Heparan-α-glucosaminide *N*-acetyltransferase (HGSNAT), the deficient enzyme in MPS IIIC, is remarkable among the enzymes deficient in MPSs in that it is the only enzyme that is not hydrolytic. HGSNAT is an acetyl transferase that catalyzes the addition of an acetyl group to the C2 amino sugar. Two fluorescent approaches—one homogeneous and one separation-based—have been described for HGSNAT measurement. The homogenous method of Voznyi et al. utilizes 4-methylumbelliferyl-β-D-glucosaminide (4-MU-βGlcN) and requires the sequential action of two enzymes: HGSNAT and β-hexosaminidase [44]. This protocol uses 4-MU-βGlcN and the cofactor acetyl-CoA (acetyl coenzyme A) in the first reaction, which is performed at pH 5.5. The second enzymatic reaction utilizes hexosaminidase and recombinant *N*-acetyl transferase to liberate 4-MU. The reactions are terminated by addition of 2-amino-2-methyl-1-propanol at pH 10.8 and fluorescence is measured at 360 nm/460 nm. The separation-based method for HGSNAT was recently described by Choi et al. [64]. This novel direct method to assay HGSNAT enzymatic activity uses fluorescent boron-dipyrromethene (BODIPY)-glucosamine as a substrate. The specificity of the assay was demonstrated using cultured fibroblasts of MPS IIIC patients, which showed profound enzymatic deficiency [64].

### 5.3. Current Challenges in MPS NBS Assays

#### 5.3.1. Matrix Challenges and Interference

Although cultured skin fibroblasts, leukocytes, plasma, or serum are the gold standard samples for diagnosis of enzymatic deficiencies associated with MPSs, DBS on filter paper are used worldwide for newborn screening tests. Following a positive newborn screening result in DBS, a confirmed diagnosis of MPS is made using the patient’s leukocytes or fibroblasts and sequencing of the respective genes. There is currently a shortage of fluorescent MPS enzyme measurement assays that are optimized for use with DBS and can be directly applied for newborn screening.

There are two main sources of optical interference to consider in fluorescent NBS assays utilizing 4-MU-based substrates and DBS extracts (excitation 340–360 nm; emission at 460 nm). The majority of optical interference in such assays comes from particulate and heme in the DBS extract itself. The 4-MU substrate can also contribute intrinsic background fluorescence, however, this is very small and is dependent on the structure and concentration of the substrate used [43,65,66]. Very pure substrates with minimal background fluorescence can be achieved by working closely with vendors who use rigorous optical methods to differentiate chemical from fluorescence impurity. To compensate for any background signal and matrix interference, two samples, one with the substrate added initially and one where it is added at the end, are used for 4-MU assays in blood samples. Hemoglobin interferes through inner filter effects that reduce the excitation intensity as well as absorb some of the emitted light in addition to some autofluorescence. Because heme levels are not the same in all samples, dual measurements with the same sample are compensated mathematically. This is less pronounced in microfluidic devices, where the path lengths are smaller. Additionally, for fluorescence substrates that are at higher wavelengths than 4-MU, optical interference from hemoglobin is avoided. Several other approaches have been employed, including the addition of trichloroacetic acid to precipitate proteins and heme, and alter the reaction pH [67]; absorption-based optical correction; and analysis of two samples with and without 4-MU with the substrates in both sets for subtraction [68].

#### 5.3.2. Substrate Specificity

Specificity is a known challenge for enzyme activity assays due to historical limitations in enantiomeric and chemical purity resulting from the substrate synthesis process (as with substrates for IDUA and GUSB, shown in Figure 3). Significant advances in methods for custom substrate synthesis and purification have enabled the production of more specific fluorescent MPS substrates at lower cost.

The common actions of many of the MPS enzymes, for example the sulfatases, also limit specificity of MPS screening assays. The substrate 4-MUS only has the sulfate group and therefore can theoretically identify deficiency in any of the 17 sulfates encoded by the human genome. Although 4-MUS is readily available, the use of this substrate necessitates significant assay optimization to differentiate activity of the sulfatase of interest from the 16 other sulfatase enzymes (Figure 6). Some specificity can be imparted to 4-MUS through the use of specific assay conditions, for example, low pH reaction conditions mimicking the lysosome restrict the assay to only the six lysosomal sulfatases. Specialized assay conditions have also been utilized to impart specificity for identification of specific sulfatase deficiencies, particularly to differentiate ARSA from ARSB when using 4-MUS as a substrate [69,70]. Ullal et al. described a novel approach for ARSB (deficient in MPS VI) in DBS that uses the 4-MUS substrate. Specificity of their approach was demonstrated via successful discrimination of clinical samples from MPS II- and MPS VI-deficient patients [71].

Finally, recent advances in second tier biochemical testing and second tier sequencing methods have made these approaches faster, more affordable, and compatible with DBS samples [72]. Second tier methods do not alter the specificity of the enzyme assay itself, but rather complement existing enzyme assays. Public health laboratories are increasingly utilizing second tier testing to identify pseudodeficiency alleles and multiple sulfatase deficiency in screen-positive samples [73].

#### 5.3.3. Multiplexing Two or More Enzymes

In the public health laboratory setting, where testing volumes are very high and the cost of each assay must be very low, it is highly desirable to multiplex NBS assays such that several analytes are interrogated from the same DBS specimen. Three approaches are currently available to support multiplexed testing of enzymatic activity: DMF-F with spatially separated multi-analyte analysis, MS/MS multiplexing by molecular weight of the mass tags, and tandem fluorescence of two or more enzymes in the same reaction vessel using fluorescently unique tags.

In DMF-F enzyme analysis, the blood sample from the DBS is extracted using a standard multi-well plate procedure before samples are loaded onto the DMF cartridge [39]. Each DMF cartridge can process 44 samples, and typically one 96-well microtiter plate of DBS extracts is used to fill the sample wells of two DMF cartridges. Once initiated, the automated cartridge protocol dispenses multiple discrete droplets of each of the blood samples, which are each combined on the cartridge with the reagent mixture for the enzyme(s) of interest. The current iteration of the DMF cartridge contains five reagent wells, and the cartridge therefore has the capability to simultaneously analyze up to five different reaction mixtures in a single cartridge run, with each reaction droplet containing the respective substrates at the appropriate pH in the presence of detergent and cofactors and/or inhibitors. Product formation is measured on the basis of the fluorescent output of each substrate after incubation and is reported as concentration of product/unit time (micromoles/liter/hour) [39].

MS/MS, like fluorescence, uses labeled enzymatic substrates to measure enzymatic activity. MS/MS tags are based on mass and enable the enzymatic reactions to be combined and measured within a single reaction vessel, although some compromise has to be made in optimal reaction conditions. Multiplexed MS/MS tests for enzyme activity can originate with either an extract from the DBS punch, as described previously for DMF-F, or can begin directly from the dry DBS punch [74,75]. Six or more enzymes have been analyzed in the same reaction using MS/MS [76]. The chemical reactions for each enzyme are all run in the same reagent mixture using a common pH (around pH 4.7), additives, and buffers. Following termination of the reaction, usually with an organic solvent, the products are extracted in the solvent, then analyzed by electrospray ionization mass spectrometry by direct flow injection, or more commonly by high-performance liquid chromatography (HPLC) prior to introduction into the MS. Although discrimination of products by mass using MS/MS inherently provides more flexibility for multiplexing, not all enzymes can be successfully combined in a single reaction mixture. When this is the case, it is possible to perform the enzymatic reactions separately under their individually optimized conditions, then combine the products prior to analysis on the mass spectrometer [33,75,77].

True multiplexed enzyme testing is also possible with fluorescence through the use of different dyes as aglycone labels. Fluorescence multiplexing is not a novel concept as it is routinely applied in protein and nucleic acid multiplexing where up to 500-plex platforms are commercially available [78]. Though this mode of fluorescence multiplexing is different, the theory for multiplexing enzyme reactions with fluorescence readout is similar to protocols used for multiplexed testing in flow cytometry or nucleic acid-sequencing techniques that are designed with complementary fluorophores of different excitation/emission wavelengths. With this experimental set-up, multiple enzymatic substrates with similar optimal reaction conditions can be incubated and detected together. We recently demonstrated a multiplexed fluorescence assay for α-galactosidase (GLA) and GLB1 within the same droplet, in which the substrates were conjugated to 4-MU and resorufin dyes, respectively (unpublished results). Resorufin is a long wavelength fluorophore (excitation 530 nm; emission at 590 nm) that, like 4-MU, has a phenolic group that can serve as the aglycone moiety when conjugated to sugars to provide enzyme-cleavable substrates. The two assays were successfully run under multiplexed conditions (within the same droplet) on the DMF-F system, with no cross-over detected between the two assays. Both of these assays have translational potential for NBS of MPS IVB (GLBI) and Fabry disease (GLA). Although resorufin substrates have many qualities that make them well-suited for enzyme activity measurement in applications such as newborn screening [79], their use is currently limited due to lack of commercial availability.

#### 5.3.4. Slow Enzymes

The normal *in vivo* activity of the enzymes deficient in the MPSs varies widely and, consequently, in vitro measurement of these enzymes will also necessitate a broad range of incubation times. The incubation times necessary for in vitro enzyme measurement can be calculated on the basis of the following assumptions. The amount of product formed is proportional to the concentration of the enzyme, the *k*_cat_ (the amount of substrate molecule each enzyme converts to product per unit time), and the incubation time of the reaction. Product formation over time is also maximized by using an excess amount of substrate (at least a 2× multiple of *K*_M_ for *V*_max_) [80]. When substrate or enzyme concentrations are limited, the reactions need to run longer to get sufficient product to compute enzymatic activity, overnight in many cases [81]. Newborn screening laboratories are accustomed to overnight assays, and this issue may be mitigated with appropriate choice of workflow. 

### 5.4. MPS Newborn Screening Using GAGs

In each of the MPS disorders, the disease phenotype is caused by aberrant catabolism of GAGs; intra-lysosomal accumulation of the non-degraded products; and subsequent cell, tissue, and organ dysfunction. Urinary GAG analysis has for many years been the gold standard for diagnosing MPS [82], however, the logical step forward to implement this approach for newborn screening is to adapt the assay for dried blood spot samples. 

Recent developments from Tomatsu *et al*. [83,84,85,86,87] and Ruitjer et al. [88] have shown feasibility for the use of GAGs in a MPS newborn screening assay using MS/MS from DBS specimens. An advantage of this method is that 10 of the 11 MPSs can be identified from a single DBS sample. Only MPS IX cannot be detected using GAG analysis due to the fact that hyaluronic acid, the GAG elevated in MPS IX, is non-sulfated. In the method described by Tomatsu et al. [86], liquid chromatography–tandem mass spectrometry (LC–MS/MS) is used to assay disaccharides derived from the four sulfated glycosaminoglycans (dermatan sulfate (DS), heparan sulfate (HS), keratan sulfate (KS), and chondroitin 6-sulfate (CS)). The GAGs are first digested with a specific enzyme to yield saccharides characteristic of the MPS disorder, separated by chromatography or electrophoresis, and finally detected by mass spectrometry. The ratio of the extracted peak area from the ion chromatograms of the disaccharides are correlated to the respective deuterated disaccharides as internal standards for quantification of the concentration of DS, HS, CS, and KS in the sample. This assay was successfully demonstrated using blood, urine, and tissues, with limited sensitivity from DBS [72,74]. Several factors have thus far prevented widespread implementation of pan-GAG analysis for multiple MPSs as a primary/first tier approach in the newborn screening setting. These include high costs for the enzymes used to digest the GAGs, the workflow burden introduced by additional sample processing steps, and fundamental requirements from the public health laboratory to report risk for each of the MPS conditions individually (reviewed in [89]). 

The more commonly used method for GAG measurement utilizes dimethyl methylene blue (DMB)—a nonspecific dye that lacks sensitivity—and is performed in urine samples, thus requiring an additional measurement of creatinine for normalization. Because fluorescence provides enhanced sensitivity to colorimetric assay readouts, approaches to detect GAGs by fluorescence have been developed. One such approach involves digesting the GAGs and labeling the end with a fluorescent dye through reductive amination [90]. Electrophoretic and chromatographic separation provides signatures that are representative of the different GAGs and has been used for MPS stratification [91]. This method is fairly cumbersome and has limited utility in a high throughput setting. Another fluorescent approach for GAG measurement is a far red dye based on the pyrene fluorophore, which has been commercialized as Heparin red. In this method, several amino chains bind to the negatively charged sulfate groups in GAGs to yield red-shifted fluorescence [92]. Heparin red is a stoichiometric binding event with no amplification and has the non-specificity inherent in such sensors. 

The pioneering work by Tomatsu et al. [72,73,74,79,82,83,84,85] has not only laid the foundational basis for the potential clinical application of GAG-based NBS for MPSs, but has also introduced a novel approach for monitoring of GAG levels in patients undergoing treatment for MPSs. As more MPS patients are identified through NBS, the demand for resources for long-term treatment—including recurrent monitoring of GAG levels and assessment of therapeutic efficacy of drugs under development—is also anticipated to rise. Emerging GAG assays utilizing MS/MS and fluorescence can be utilized with blood rather than urine samples and offer a promising alternative to DMB or Alcian blue methods for GAG analysis [93,94,95]. 

## 6. Other Considerations for NBS of MPS

Although the benefits of NBS are clearly documented by the improved clinical outcomes in affected babies who receive early diagnosis and treatment, there are certain risks associated with MPS NBS that can impact newborns and their families. The most common risk associated with MPS NBS is the emotional stress to the family of infants receiving false-positive test results [96,97]. These families may be recalled to a follow-up testing center where the baby undergoes additional biochemical, physiological, and/or molecular testing to determine a disease diagnosis. As the clinical phenotypes of each MPS are highly heterogeneous, the severity of the clinical phenotype can be difficult to determine in infants with confirmed MPS [96,97,98]. Molecular testing is of great benefit to MPS NBS as it enables timely identification of pseudodeficiency variants (variants causing low enzyme activity in the screening assay, but no overt disease phenotype) and heterozygous carriers. There are however also a growing number of variants of uncertain significance (VUS) and compound cases of heterozygous pathogenic mutations together with one or more VUS for the various MPS in the literature [99]. Clinical management of such cases is challenging because the onset of disease phenotype is unclear. Such diagnoses also cause significant anxiety for families of affected infants. In addition to the technical aspects previously presented, it is also important that each newborn screening program considering adding a MPS to their panel carefully evaluate the ethical and psychological impact of screening.

## 7. Conclusions

As more MPS therapies are developed and approved, the demand for efficient, cost effective newborn screening methods is anticipated to grow. Methods that are compatible with multiplexing will enable more babies to be screened for more of the MPS subtypes and will thereby have the greatest clinical impact. Fluorescence-based newborn screening assays provide an excellent option by combining simple, highly specific reaction conditions with a detection method that is accessible to even low-resourced laboratories. The inherent flexibility of fluorescence enables this detection modality to be applied in a variety of settings. Fluorescent MPS newborn screening assays have been miniaturized using digital microfluidics technology, and future maturation of this technology has the potential to bring MPS assays out of the centralized laboratory and closer to the patient for diagnostic monitoring during treatment. For patients already diagnosed with an MPS and undergoing treatment, fluorescence-based assays also have the potential to be used to monitor the impact of treatments via rapid measurement of GAG levels in blood. Furthermore, fluorescent aglycone substrate moieties can be analyzed using mass spectrometry and provide an alternative to deuterated calibrants.

## Figures and Tables

**Table 1:** Summary of MPS subtypes with published fluorescent screening assays available.

MPS	OMIM#	Gene	Deficient Enzyme (EC#)	Elevated GAG	Key Disease Features	FDA-Approved Therapies
MPS I (Hurler, Scheie, Hurler/Scheie)	607014, 607015, 607016	*IDUA*	α-iduronidase (3.2.1.76)	DS, HS	Corneal clouding, skeletal abnormalities, organ enlargement, heart disease, mental retardation, death in childhood	Aldurazyme
MPS II (Hunter)	300823	*IDS*	α-iduronide sulfatase (3.1.6.13)	DS, HS		Elaprase
MPS IIIA (Sanfilippo A)	252900	*SGSH*	*N*-sulfoglucosamine sulfohydrolase (3.10.1.1)	HS	Profound mental deterioration, hyperactivity, and mild somatic manifestations	
MPS IIIB (Sanfilippo B)	252920	*NAGLU*	Acetyl α-glucosaminidase (3.2.1.50)	HS		
MPS IIIC (Sanfilippo C)	252930	*HGSNAT*	α-glucosaminide *N*-acetyl transferase (2.3.1.78)	HS		
MPS IIID (Sanfilippo D)	252940	*GNS*	*N*-acetyl glucosamine-6-sulfatase (3.1.6.14)	HS		
MPS IVA (Morquio A)	253000	*GALNS*	*N*-acetyl galactosamine -6-sulfatase (3.1.6.4)	KS, CS	Skeletal abnormalities, loose ligaments, degenerative joint disease, corneal clouding, heart disease, death in childhood or young adulthood	Vimzim
MPS IVB (Morquio B)	253010	*GLB1*	β-galactosidase (3.2.1.23)	KS		
MPS VI (Maroteaux-Lamy)	253200	*ARSB*	*N*-acetyl galactosamine 4-sulfatase (3.1.6.1)	DS, CS	Corneal clouding, skeletal abnormalities, organ enlargement, heart disease, death in childhood	Naglazyme
MPS VII (Sly)	253220	*GUSB*	β-glucuronidase (3.2.1.31)	DS, HS, CS	Corneal clouding, skeletal abnormalities, organ enlargement, heart disease, mental retardation, death in childhood	Mepsevii

GAG abbreviations: dermatan sulfate (DS), heparan sulfate (HS), keratan sulfate (KS), chondroitin sulfate (CS).

**Table 2:** Summary of the fluorescent substrates, assay formats, and samples analyzed using MPS enzyme activity assays.

Condition	Fluorescent Substrate	Assay	Sample Types
**MPS I**	4MU-α-l-iduronide (4MU-IdoA)	1 step + pH change	DBS, recombinant (r)IDUA, fibroblasts, leukocytes
**MPS II**	4MU-α-l-iduronide-2-sulfate (4MU-IdoA2S)	2 steps + pH change	DBS, rIDS, fibroblasts, leukocytes
**MPS IIIA**	4MU-N-sulpho-α-d-glucosaminide (4MU-Glc2NS)	2 steps + pH change	rSGSH, fibroblasts, leukocytes, cell lysates
**MPS IIIB**	4MU-N-acetyl-α-d-glucosaminide (4MU-GlcNAc)	1 step + pH change	DBS, rNAGLU, fibroblasts, leukocytes
**MPS IIIC**	4MU-2-amino-deoxy glucose (4MU-GlcN)	2 steps + pH change	rHGSNAT, leukocytes, fibroblasts, cell lysates
**MPS IIID**	4MU-N-acetyl-α-d-glucosaminide-6-sulfate (4MU-GlcNAcS)	2 steps + pH change	rGNS, fibroblasts, leukocytes
**MPS IVA**	4MU-N-acetyl-α-d-galactoseaminide-6-sulfate (4MU-GalNAc-6S)	2 steps + pH change	rGALNS, DBS, leukocytes
**MPS IVB**	4MU-β-d-galactoside (4MU-Gal)	1 step + pH change	rβGal, DBS
**MPS VI**	4MU-N-acetyl-α-d-galactoseaminide-4-sulfate (4MU-GalNAc-4S)	2 steps + pH change	rARSB, DBS
	4MUS	1 step + pH change	
**MPS VII**	4MU-β-d-glucuronide (4MU-GlcA)	1 step + pH change	rβGlc, DBS

Red font in the substrate column indicates a sulfate group linked to the saccharide moiety. Enzyme abbreviations were defined in Table 1. 4MU: 4-methylumbelliferyl; DBS: dried blood spots.

## Abbreviations

4-MU	4-methylumbelliferone
DMF	digital microfluidics
DMF-F	digital microfluidics fluorescence
NBS	newborn screening
LSD	lysosomal storage disorder
GAG	glycosaminoglycan
DBS	dried blood spot
MPS	mucopolysaccharidosis
IDUA	α-iduronidase
IDS	α-iduronide sulfatase
SGSH	*N*-sulfoglucosamine sulfohydrolase
NAGLU	acetyl α-glucosaminidase
HGSNAT	α-glucosaminide *N*-acetyl transferase
GNS	*N*-acetyl glucosamine -6-sulfatase
GALNS	*N*-acetyl galactosamine -6-sulfatase
GUSB	β-glucuronidase
β-gal	β-galactosidase
GLB1	β-glucuronidase
ARSB	*N*-acetyl galactosamine 4-sulfatase
ARSA	arylsulfatase A
Ido	iduronate
CS	chondroitin sulfate
DS	dermatan sulfate
HS	heparan sulfate
KS	keratin sulfate
MS/MS	tandem mass spectrometry
LC–MS/MS	liquid chromatography–tandem mass spectrometry
